# Seminal plasma biomarkers for predicting successful sperm retrieval in patients with nonobstructive azoospermia: a narrative review of human studies

**DOI:** 10.1186/s12610-023-00184-0

**Published:** 2023-04-20

**Authors:** Junjun Li, Fang Yang, Liang Dong, Degui Chang, Xujun Yu

**Affiliations:** 1 Chengdu Fifth People’s Hospital, The Fifth People’s Hospital of Chengdu University of Traditional Chinese Medicine, 611130 Chengdu, China; 2 TCM Regulating Metabolic Diseases Key Laboratory of Sichuan Province, Hospital of Chengdu, University of Traditional Chinese Medicine, 610072 Chengdu, China; 3 The Reproductive & Women-Children Hospital, Chengdu University of Traditional Chinese Medicine, 610041 Chengdu, China; 4 School of Medical and Life Sciences, Chengdu University of Traditional Chinese Medicine, 611137 Chengdu, China

**Keywords:** Male infertily, Non-obstructive azoospermia, Surgical testicular sperm extraction, Successful sperm retrieval, Seminal plasma, Infertilité masculine, Azoospermie non obstructive, Extraction chirurgicale de Spermatozoïdes testiculaires, Récupération réussie de Spermatozoïdes, Plasma séminal

## Abstract

**Background:**

Non-obstructive azoospermia (NOA) is considered to be the most severe form of male infertility. Before the emergence of surgical testicular sperm extraction and assisted reproductive technology, NOA patients could hardly become biological fathers of their children. However, failure of the surgery could cause physical and psychological harm to patients such as testicular damage, pain, hopeless of fertility and additional cost. Therefore, predicting the successful sperm retrieval (SSR) is so important for NOA patients to make their choice whether to do the surgery or not. Because seminal plasma is secreted by the testes and accessory gonads, it can reflect the spermatogenic environment, making it a preferential choice for SSR valuation. The purpose of this paper is to summarize the available evidence and provide the reader with a broad overview of biomarkers in seminal plasma for SSR prediction.

**Results:**

A total of 15,390 studies were searched from PUBMED, EMBASE, CENTRAL and Web of Science, but only 6615 studies were evaluated after duplications were removed. The abstracts of 6513 articles were excluded because they were irrelevant to the topic. The full texts of 102 articles were obtained, with 21 of them being included in this review. The included studies range in quality from medium to high. In the included articles, surgical sperm extraction methods included conventional testicular sperm extraction (TESE) and microdissection testicular sperm extraction (micro-TESE). Currently, the biomarkers in seminal plasma used to predict SSR are primarily RNAs, metabolites, AMH, inhibin B, leptin, survivin, clusterin, LGALS3BP, ESX1, TEX101, TNP1, DAZ, PRM1 and PRM2.

**Conclusion:**

The evidence does not conclusively indicate that AMH and INHB in seminal plasma are valuable to predict the SSR. It is worth noting that RNAs, metabolites and other biomarkers in seminal plasma have shown great potential in predicting SSR. However, existing evidence is insufficient to provide clinicians with adequate decision support, and more prospective, large sample size, and multicenter trials are urgently needed.

## Résumé

**Contexte:**

L’azoospermie non obstructive (ANO) est considérée comme la forme la plus grave d’infertilité masculine. Avant l’émergence de l’extraction chirurgicale de spermatozoïdes testiculaires et de la procréation médicalement assistée, les patients atteints d’ANO pouvaient difficilement devenir les pères biologiques de leurs enfants. Cependant, l’échec de la chirurgie peut causer des dommages physiques et psychologiques aux patients, tels que des lésions testiculaires, des douleurs, une fertilité impossibilité et des coûts financiers supplémentaires. Par conséquent, prédire une récupération réussie de spermatozoïdes (RRS) est très important pour les patients avec ANO, pour faire leur choix de réaliser ou non la chirurgie. Comme le plasma séminal est sécrété par les testicules et les glandes accessoires, il peut être le reflet de l’environnement de la spermatogenèse, ce qui en fait un choix préférentiel pour l’évaluation de la RRS. Le but du présent article est de résumer les preuves disponibles, et de fournir au lecteur un aperçu général des biomarqueurs du plasma séminal susceptibles de prédire la RRS.

**Résultats:**

Au total, 15390 études ont été  consultées à partir de PUBMED, EMBASE, CENTRAL et Web of Science, mais seulement 6615 ont été évaluées après suppression des doublons. Les résumés de 6513 articles ont été exclus car non pertinents pour le sujet. Les textes intégraux de 102 articles ont été obtenus, dont 21 ont été inclus dans cette revue. La qualité des études incluses varie de moyenne à élevée. Dans ces articles inclus, la méthode chirurgicale d’extraction de spermatozoïdes était soit l’extraction conventionnelle de spermatozoïdes testiculaires (TESE), soit l’extraction de spermatozoïdes testiculaires par microdissection (micro-TESE). Actuellement, les biomarqueurs du plasma séminal utilisés pour prédire la RRS sont principalement les ARN, les métabolites, l’AMH, l’inhibine B, la leptine, la survivine, la clusterine, LGALS3BP, ESX1, TEX101, TNP1, DAZ, PRM1 et PRM2.

**Conclusions:**

Les preuves n’indiquent pas de façon concluante que l’AMH et l’INHB dans le plasma séminal sont utiles pour prédire la RRS. Il convient de noter que les ARN, métabolites et autres biomarqueurs du plasma séminal ont montré un potentiel élevé de prédiction de la  RRS. Cependant, les preuves existantes sont insuffisantes pour fournir aux cliniciens une aide à la décision adéquate ; des essais plus prospectifs, de grande taille, et multicentriques sont nécessaires de toute urgence.

## Introduction

It is estimated that infertility affects 8–12% of couples globally, with a male factor being a primary or contributing cause in approximately 50% of couples [1, 2]. Non-obstructive azoospermia (NOA) is defined as the absence of sperm in ejaculation caused by impaired spermatogenesis, which is the most severe form of male infertility. The prevalence of NOA is 1% in all men and 10-15% in infertile men [3–5]. Historically, NOA men are considered to be infertile, but the emergence of testicular sperm extraction and ICSI has made it possible for NOA men to become biological fathers of their children. Although the testicular sperm extraction technology is constantly improving, about 50% of NOA patients still cannot find sperm through surgery, and surgery will inevitably lead to testicular damage and increase the pain and cost consumption of patients. Therefore, how to determine whether it is necessary to carry out testicular sperm extraction through preoperative evaluation is a problem that reproductive andrologists have been exploring. Successful sperm retrieval (SSR) is defined as obtaining at least one spermatozoon via surgical testicular sperm extraction [6]. Although the value of serum hormonal markers in predicting SSR was expected, the research results were unsatisfactory [7].

Seminal plasma (SP) is a biological fluid made up of secretions from glands, such as testes, in the male urogenital tract. It could be a rich source of noninvasive biomarkers, including tissue-specific RNAs and proteins, where abnormal changes could directly indicate a pathological process in the testis [8]. Therefore, the utility of SP biomarkers in predicting SSR is constantly being investigated. To date, the genomic, transcriptomic, proteomic, and metabolomic profiles of human SP have been studied to predict SSR, with promising results [9]. This review aims to summarize the differences in SP biomarkers in NOA men with positive and negative surgical TESE outcomes and to provide the reader with a broad overview of SP biomarkers for predicting SSR.

## Material and methods

A search of PUBMED, EMBASE, CENTRAL and Web of Science were made for studies that compared SP biomarkers in NOA men with positive and negative surgical testicular sperm extraction (Table 1). The search terms considered the following MeSH (Medical Subject Headings) terms, which were adjusted for each database: (Semen OR “Seminal Plasma” OR “Plasma, Seminal” OR “Seminal fluid” ) AND (Azoospermia OR Aspermia). No language or date limits were set. Studies that compared biomarkers in SP of patients with NOA and patients with Obstructive Azoospermia (OA) or oligospermia or normozoospermia were excluded because these studies were not direct evidence to predict SSR. Only studies that compared SP biomarkers in NOA men with positive and negative outcomes of surgical testicular sperm extraction were included. Newcastle-Ottawa scale (NOS) was used to evaluate the quality assessment of the enrolled nonrandomized studies [10]. The quality assessment of the selected studies was independently completed by two reviewers (Junjun Li and Xujun Yu). Studies were then classified according to their quality into poor (0–4), moderate (5–6), or high quality (7–9).

**Table 1:** Search strategy summary

Items	Specification
Date of search	12/1/2022
Databases and other sources searched	PUBMED, EMBASE, CENTRAL and Web of Science.
Search terms used	(Semen OR “Seminal Plasma” OR “Plasma, Seminal” OR “Seminal fluid” ) AND (Azoospermia OR Aspermia)
Timeframe	No limit
Inclusion and exclusion criteria	Inclusion criteria: Studies that compared SP biomarkers in NOA men with positive and negative outcomes of surgical testicular sperm extraction.Exclusion criteria: Studies that compared biomarkers in SP of patients with NOA and patients with OA or oligospermia or normozoospermia.
Selection process	Selection performed by Junjun Li and Xujun Yu.

*NOA* non-obstructive azoospermia, *OA *obstructive azoospermia

## Results

**Fig. 1:**
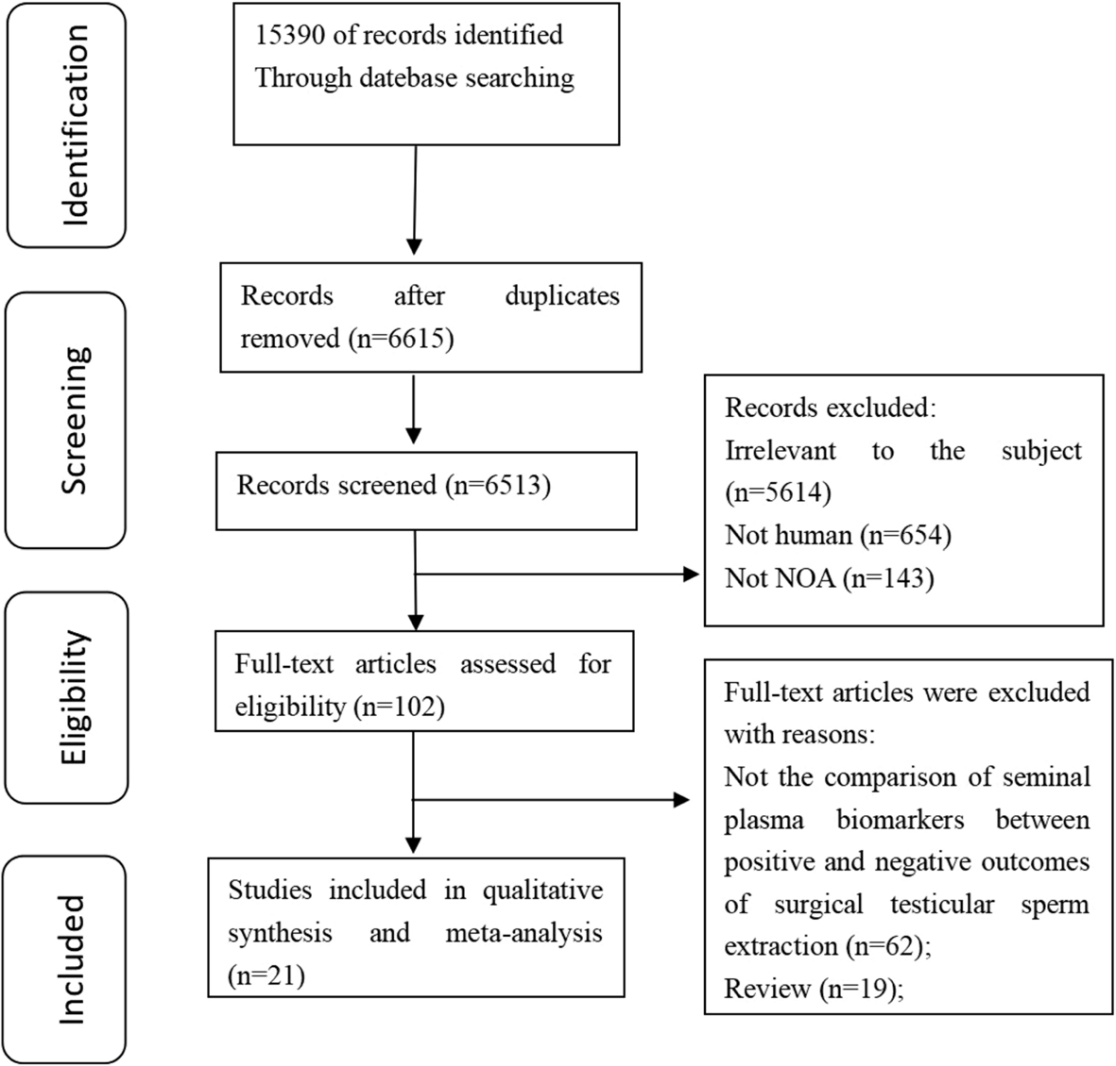
Flowchart of study selection process. A total of 15,390 studies were searched, but only 6615 studies were evaluated after duplications were removed. The abstracts of 6513 articles were excluded because they were irrelevant to the topic. The full texts of 102 articles were obtained, with 21 of them being included in this review

A total of 15,390 studies were searched, but only 6615 studies were included for evaluation after duplications were removed. 6513 articles were excluded based on the abstracts irrelevant to the topic. The full texts of 102 articles were retrieved, of which 81 articles did not meet the inclusion criteria and were excluded from the review (Fig. 1). Therefore, a total of 21 articles [11–31] were included in this review. One study did not mention the surgical sperm extraction method [11], 13studies [12–20, 23–26] compared the difference of SP biomarkers between positive and negative conventional testicular sperm extraction (TESE) outcomes, and six studies [22, 27–31] compared the difference of SP biomarkers between positive and negative microdissection testicular sperm extraction (micro-TESE) outcomes in NOA men. One study studied both TESE and micro-TESE [21]. Among the SP biomarkers investigated are RNAs, metabolites, AMH, inhibin B and others (See Table 2 ). According to the NOS for nonrandomized studies, the quality assessment was considered moderate [11, 12, 14, 15, 19–26, 30] and high quality [13, 17, 18, 27–29, 31], as demonstrated in Table 3.

**Table 2:** Seminal plasma biomarkers for predicting successful sperm retrieval in men with nonobstructive azoospermia

Year	Authors	Sample size	Predictors	Results	Country
1999	Fénichel et al. [11]	10 NOA men with positive surgical sperm extraction and 13 NOA men with negative surgical sperm extraction.	AMH	In NOA group, comparison of seminal AMH concentration and the results of histological analysis of testicular biopsies revealed that undetectable AMH found in 14 cases was associated in 11 of them with lack of spermatozoa, whereas detectable concentrations of AMH (10–68.5 pmol/L) found in nine cases were associated in seven of them with persistent spermatogenesis (no surgical method provided).	France
2005	Nagata et al. [12]	17 NOA men with TESE (+) and 45 NOA men with TESE (-).	INHB	The seminal inhibin B concentrations were significantly higher in the TESE (+) group compared with the TESE (−) group.	Japan
2007	Mostafa et al. [13]	19 NOA men with TESE (+) and 21 NOA men with TESE (-).	AMH	There were no significant differences in terms of AMH between TESE (+) and TESE (−) patients.	Egypt
2008	Duvilla et al. [14]	11 NOA men with TESE (+) and 15 NOA men with TESE (-).	AMH and INHB	There were no significant differences in terms of AMH and inhibin B between TESE (+) and TESE (−) patients.	France
2009	Roshdy et al. [15]	24 NOA men with TESE (+) and 13 NOA men with TESE (-).	Survivin	Seminal survivin was detectable in TESE (+) men but not in TESE (−) men.	Egypt
2010	Sabetian et al. [16]	8 NOA men with TESE (+) and 70 NOA men with TESE (-).	fructose, NAG, INHB, and AMH	There were significant differences in inhibin B and AMH concentrations between TESE (+) and TESE (−) patients. There were no differences regarding the levels of NAG and fructose between TESE (+) and TESE (−) patients.	Iran
2010	Mitchell et al. [17]	60 NOA men with TESE (+) and 79 NOA men with TESE (-).	AMH and INHB	TESE (+) and TESE (−) patients did not differ significantly in terms of either AMH or inhibin B seminal plasma concentrations.	France
2011	Ma et al. [18]	110 NOA men with TESE (+) and 170 NOA men with TESE (-).	leptin, alpha-glucosidase and fructose	There was a significant difference in SP-leptin levels between positive and negative SSR groups, and the cut-off value was 2.9 ng/mL (sensitivity of 43.1% and specificity of 75.0%) to predict SSR at TESE with 59% accuracy. Combined leptin and other markers can significantly improve the prediction accuracy of sperm retrieval in NOA patients. There were no significant differences in terms of seminal α-glucosidase and fructose between TESE (+) and TESE (−) patients.	China
2011	Aslani et al. [19]	21 NOA men with TESE (+) and 89 NOA men with TESE (-).	PRM1, PRM2, AKAP4, and DAZ	Comparison of the biomarkers and TESE (+) results did not show any significant correlation. But the presence of germ cell-specific DAZ and PRM2 transcripts in the semen of NOA patients can be used as specific noninvasive markers to predict the presence of mature spermatids and/or sperm in the testis.	Germany
2013	Freour et al. [20]	20 NOA men with TESE (+) and 20 NOA men with TESE (-).	LGALS3BP	Seminal LGALS3BP expression was found to be significantly higher in TESE (+) men.	France
2014	Pansa et al. [21]	20 NOA men with TESE (+) and 16 NOA men with TESE (-);24 NOA men with micro-TESE (+) and 18 NOA men with micro-TESE (-).	ESX1	ESX1 mRNA expression in the testis was strongly correlated with TESE (+) and micro-TESE (+).	Italy
2016	Fukuda et al. [22]	9 NOA men with micro-TESE (+) and 19 NOA men with micro-TESE (-).	clusterin	There were no significant differences in terms of clusterin between micro-TESE (+) and micro-TESE (−) patients. However, univariate analysis identified seminal clusterin level as significant predictors of SSR by micro-TESE.	Japan
2017	Korbakiset al. [23]	15 NOA men with TESE (+) and 11 NOA men with TESE (-)	TEX101	A cut-off value of ≥ 0.6 ng/mL provided 73% sensitivity at 64% specificity for predicting sperm or spermatid retrieval in patients with NOA by TESE.	Canada
2017	Gilany et al. [24]	9 NOA men with TESE (+) and 11 NOA men with TESE (-)	metabolites	Thirty-six differentiating metabolites were identified as prognostic biomarkers for TESE (−) and TESE (+) men.	Iran
2018	Gilany et al. [25]	10 NOA men with TESE (+) and 10 NOA men with TESE (-).	metabolic fingerprinting	Metabolic fingerprint, particularly oxidative status, significantly differed between TESE (+) and TESE (−) patients.	Iran
2018	Barceló et al. [26]	8 NOA men with TESE (+) and 4 NOA men with TESE (-).	microRNAs	No significant differences between TESE (+) and TESE (−) were found regarding the individual levels of the eight miRNAs and the piRNA in SP exosomes. However, when the multivariate regression analysis was performed, it resulted in a model that included the miR-539-5p and the miR-941 expression values.	Spain
2020	Hashemi et al. [27]	8 NOA men with micro-TESE (+) and 11 NOA men with micro-TESE (-)	ESX1, ZMYND15, TNP1 and PRM1	No significant differences between micro-TESE (−) and micro-TESE (+) were found regarding the level of ESX1 and ZMYND15. However, the expression level of TNP1 and PRM1 was significantly decreased in the sample with micro-TESE (−) versus micro-TESE (+).	Iran
2020	Xie et al. [28]	64 NOA men with micro-TESE(+) and 32 NOA men with micro-TESE(-)	long noncoding RNAs	A biomarker panel of lncRNAs (LOC100505685, SPATA42, CCDC37-DT, GABRG3-AS1, LOC440934, LOC101929088 (XR_927561.2),LOC101929088 (XR_001745218.1), LINC00343 and LINC00301)was constructed and verified in 64 NOA patients with micro-TESE (+) and 32 NOA patients with micro-TESE (−). When the decision-making process score exceeds 0.532 (cut-off value), the predictive accuracy of our panel reaches 95.238%, and the sperm retrieval surgery is recommended.	China
2021	Ji etal. [29]	20 NOA men with micro-TESE(+) and 32 NOA men with micro-TESE(-)	circRNAs	circRNAs (hsa_circ_0000277, hsa_circ_0060394 and hsa_circ_0007773) significantly differed between micro-TESE (+) and micro-TESE (−) patients.	China
2022	Zhang et al. [30]	53 NOA men with micro-TESE(+) and 23 NOA men with micro-TESE(-).	microRNAs	A biomarker panel of microRNAs (hsa-miR-34b-3p, hsa-miR-34c-3p, hsa-miR-3065-3p, and hsa-miR-4446-3p) was constructed and tested in 53 NOA patients with micro-TESE (+) and 23 NOA patients with micro-TESE (−). The predictive model provided a high predictive accuracy (AUC = 0.927).	China
2022	Han et al. [31]	18 NOA men with micro-TESE(+) and 23 NOA men with micro-TESE(-).	tsRNAs	The extracellular vesicle tRF-Val-AAC-010 resulted in high predictive ability (AUC = 0.89, sensitivity = 72%, specificity = 91%, *P* < 0.0001) for predicting the presence of sperm in nonobstructive azoospermia undergoing micro-TESE.	China

(+), successful; (-), unsuccessful; *AMH *Anti-Mullerian hormone, *INHB* Inhibin B, *NOA* Non-obstructive azoospermia, *NAG* neutral alpha glucosidase, *PRM1* Protamine 1, *PRM2* Protamine 2, *AKAP4* A-kinase anchoring protein-4, *DAZ* Deleted in azoospermia, *LGALS3BP* Lectin galactoside-binding, soluble 3 binding protein, *ESX1* Extra-embryonic tissue-spermatogenesis-homeobox gene 1, *TEX101* Testis expressed gene 101, *TNP1* Transition Protein 1, *ZMYND15* Zinc Finger MYND Domain-Containing Protein 15

**Table 3:** Newcastle-Ottawa Scale for assessing the quality of the included studies

Authors	Is the case definition adequate?	Representativeness of the cases.	Selection of controls	Definition of controls	Comparability of cases and controls on the basis of the design or analysis	Ascertainment of exposure	Same method of ascertainment for cases and controls	Non-response Rate	Total scores
Fénichel et al. [11]	☆	☆	☆	☆	☆	☆	-	-	6
Nagata et al. [12]	☆	-	☆	-	☆☆	☆	☆	-	6
Mostafa et al. [13]	☆	☆	☆	☆	☆	☆	☆	-	7
Duvilla et al. [14]	☆	☆	☆	-	☆	☆	☆	-	6
Roshdy et al. [15]	☆	-	☆	☆	☆	☆	☆	-	6
Sabetian et al. [16]	☆	☆	☆	-	☆	☆	☆	-	6
Mitchell et al. [17]	☆	☆	☆	☆	☆	☆	☆	-	7
Ma et al. [18]	☆	☆	☆	☆	☆☆	☆	☆	-	8
Aslani et al. [19]	☆	-	☆	-	☆	☆	☆	-	5
Freour et al. [20]	☆	☆	☆	-	☆	☆	☆	-	6
Pansa et al. [21]	☆	-	☆	-	☆	☆	☆	-	5
Fukuda et al. [22]	☆	-	☆	-	☆☆	☆	☆	-	6
Korbakiset al. [23]	☆	☆	☆	-	☆	☆	☆	-	6
Gilany et al. [24]	☆	-	☆	-	☆☆	☆	☆	-	6
Gilany et al. [25]	☆	-	☆	-	☆☆	☆	☆	-	6
Barceló et al. [26]	☆	-	☆	-	☆	☆	☆	-	5
Hashemi et al. [27]	☆	☆	☆	☆	☆	☆	☆	-	7
Xie et al. [28]	☆	☆	☆	-	☆☆	☆	☆	-	7
Ji et al. [29]	☆	☆	☆	☆	☆☆	☆	☆	-	8
Zhang et al. [30]	☆	☆	☆	-	☆	☆	☆	-	6
Han et al. [31]	☆	☆	☆	☆	☆☆	☆	☆	-	8

## Discussion

### Seminal Plasma Transcriptome (RNAs)

The term transcriptome refers to the total amount of RNA in a specific specimen. The human SP contains approximately 700 extracellular RNAs that are involved in regulating vesicle-mediated transport, protein kinase inhibition, and cell response to zinc, which may reflect the spermatogenesis state in the testis [32]. To date, the biomarkers studied in studies of SP RNAs difference between NOA patients with positive and negative surgical sperm extraction include microRNAs [26, 30], long noncoding RNAs [28], circRNAs [29], and tsRNAs [31].

### MicroRNAs

MicroRNAs (miRNAs) are small noncoding RNA molecules with 18–25 nucleotides, which can regulate post translation gene expression by triggering translation inhibition or mRNA degradation. It has been confirmed that miRNAs are involved in the regulation of germ cell development and spermatogenesis [33, 34]. Given the abnormal expression of various miRNAs involved in spermatogenesis in NOA patients and the importance of miRNAs targeting pathways in successful spermatogenesis [35], it is reasonable to believe that miRNA has a high potential for predicting SSR. In fact, miRNAs exist not only in the intracellular environment but also in the extracellular environment, particularly in various biofuels, such as SP [36]. miRNAs are found in SP as microvesicular bodies and protein-bonded complexes [37]. Because of the existence and distribution of miRNAs, semen miRNAs are protected from degradation by existing RNase enzymes, making them potential noninvasive indicators of spermatogenesis.

Barceló et al. [26] found no significant differences in SP-miRNAs between eight NOA patients who had successful TESE and four NOA patients who had failed TESE. A multiple regression analysis model containing miR-539-5p and miR-941 had 100% sensitivity and specificity in predicting the presence of sperm in testicular biopsy for the samples in the study. Zhang et al. [30] recently examined the SP-miRNAs from NOA patients with successful and unsuccessful micro-TESE (*n* = 6 in each group) and fertile men (*n* = 6). Their results showed that high levels of hsa-miR-34b-3p and hsa-miR-34c-3p expressions were associated with successful micro-TESE. In contrast, high levels of hsa-miR-3065-3p and hsa-miR-4446-3p expressions are associated with failed micro-TESE. Furthermore, a predictive model based on these four miRNAs was developed with high predictive accuracy (AUC = 0.927). Although these SP-miRNAs need to be validated in larger cohort studies, the current results indicate that SP-miRNAs have a high potential for predicting the SSR of NOA patients.

### Long noncoding RNAs

Long non-coding RNAs (lncRNAs) are a group of RNA transcripts with longer than 200 nucleotides and without protein-coding capacity. It was revealed that the lnc RNAs could be involved in the regulation of gene expression at the transcriptional, post-transcriptional and epigenetic levels, and chromatin remodeling. Studies have also demonstrated that lncRNAs play a critical role in spermatogenesis [38, 39], indicating that lncRNAs may be potential biomarkers for predicting SSR in NOA men. Xie et al. [28] recently sequenced the RNA of SP extracellular vesicle from six normozoospermic men and five idiopathic NOA patients with failed micro-TESE. They constructed and validated a lncRNA biomarker panel in 96 NOA patients receiving micro-TESE. Then, a prediction model (AUC was 0.986 in the training set and 0.960 in the validation set) with nine differentially expressed testis-specific lncRNAs, including LOC100505685, SPATA42, CCDC37-DT, GABRG3-AS1, LOC440934, LOC101929088 (XR_927561.2), LOC101929088 (XR_001745218.1), LINC00343, and LINC00301, was established. The most important finding of this study is that in the same group of patients with NOA, the prediction ability of the model based on these nine lncRNAs for SSR is significantly higher than that based on sex hormones. Finally, sperm retrieval surgery may be recommended if the score of the NOA case exceeds 0.532, according to the decision-making procedure made by the lncRNA panel. However, the regulatory functions and mechanisms of these lncRNAs in spermatogenesis must be clarified, and their predictive value must be confirmed in larger sample size studies.

### CircRNAs

Circular RNAs (circRNAs) are covalent closed-loop structures formed by front-to-tail reverse shearing of precursor mRNAs [40].CircRNAs are stable in a variety of body fluids (such as semen and blood) because they are not affected by RNA exonuclease. CircRNAs could also be detected in seminal plasma at room temperature [41]. The role of circRNAs in spermatogenesis has been established, and several circRNAs have been found in seminal plasma, implying that circRNA deregulation is linked to various types of male infertility, including NOA [42]. Ji et al. [29] discovered three SP circRNAs (hsa_circ_0000277, hsa_circ_0060394 and hsa_circ_0007773) that differed between NOA patients with successful and unsuccessful micro-TESE, and these three circRNAs have a high predictive value for micro-TESE results (AUC values: 0.920, 0.928 and 0.891, respectively). Furthermore, a new prediction model based on the combination of the three circRNAs demonstrated good SSR prediction value (AUC value: 0.958), indicating that circRNAs derived from SP testis may be used as reliable biomarkers to predict SSR in patients with NOA. However, the number of cases in this study was small, and the function of these circRNAs and their relationship to spermatogenesis remain unknown.

### tsRNAs

tRNA derived small RNAs (tsRNAs), known as new regulatory small non coding RNAs, are precisely regulated by the processing of tRNA and its precursors. It has been demonstrated that tsRNAs are widely involved in large quantities of physiological and pathological processes [43]. According to studies, tsRNAs are closely related to mammalian sperm maturation and fertilization and are abundant in SP [44, 45], implying that tsRNAs could be potential biomarkers for SSR prediction. Han et al. [31] recently compared the SP extracellular vesicle tsRNA levels in 18 NOA patients with successful micro-TESE and 23 NOA patients with unsuccessful micro-TESE and found that tRF-Val-AAC-010 expression levels were higher in the successful micro-TESE group than in the unsuccessful micro-TESE group, resulting in good predictive accuracy (AUC = 0.89, sensitivity = 72%, specificity = 91%, *P* < 0.0001). Moreover, tRF-Val-AAC-010 had a higher predictive accuracy than testicular volume and serum FSH. More importantly, it was demonstrated that tRF-Val-AAC-010 can be expressed in testicular tissues of patients with NOA and participate in spermatogenesis, implying that tRF-Val-AAC-010 could be a potential SP biomarker for predicting SSR.

### Metabolites

Following genomics and proteomics, metabonomics is a developed discipline that is an important part of systems biology. Gene and protein expression are closely linked, whereas metabolites can better reflect the cell environment, which is closely related to cell nutrition, the role of drugs and pollutants in the environment, and other external factors. Approximately 40,000 human metabolites have been found in the 3.0 Human Metabolome Database [46]. At present, a number of metabonomic methods have been defined, including metabolome mapping, metabolic fingerprinting, metabolic profiling, metabolic footprinting, and metabolic target analysis, and untargeted metabolic profiling. The classification of specific metabolic biomarkers, in particular, is useful in better identifying male infertility [47]. Existing research suggests that metabolic fingerprinting of seminal plasma can be used as a useful diagnostic tool for men experiencing infertility [48, 49]. To the best of our knowledge, Gilany et al. [24] investigated metabolic profiling of SP of NOA men with successful and unsuccessful TESE and discovered 36 distinguishing metabolites as prognostic biomarkers for positive and negative TESE men. Furthermore, another study [25] revealed that the metabolic fingerprint, particularly the oxidative status, differed significantly between positive and negative TESE patients. These results indicate that SP metabolites have great potential as specific biomarkers for SSR prediction. The main shortcoming of these studies is that these metabolites lack effective validation and are not included in the Human Metabolome Database. Hence, more in-depth studies are required in the future.

### AMH

The Anti-Mullerian hormone (AMH), which plays an important role in the maturation and differentiation of spermatogenic cells, is expressed in Sertoli cells and it governs the regression of the Müllerian ducts of the male fetus [50]. It is worth noting that after puberty, AMH secretion into the seminiferous tubule lumen by apical Sertoli cells is greater than basal secretion into the interstitium and blood circulation. Therefore, AMH concentrations in SP are higher in adult males than in serum [51, 52]. Moreover, AMH in SP can provide direct information about spermatogenesis [53]. This suggests that when investigating the possible relationship between AMH and spermatogenesis, it is preferable to measure SP-AMH rather than serum AMH. For this reason, the importance of SP-AMH in predicting SSR in patients with NOA is emphasized.

Fénichel et al. [11] published the first study comparing the concentrations of SP-AMH between patients with NOA who failed with surgical sperm extraction and those who succeeded in 1999, and the results revealed that of the 14 cases in which AMH could not be detected, 11 were negative for surgical sperm extraction, whereas seven of the nine cases in which AMH was detected were positive for surgical sperm extraction, indicating that AMH in SP is related to the SSR. However, the method of surgical sperm extraction was not mentioned in this study, and the sample size was only 23. Sabetian et al. [16] also reported that SP-AMH concentrations differed significantly between successful and unsuccessful TESE NOA men. Other studies came to the opposite conclusions as a result. Mostafa et al. [13] found that in the 17 cases where AMH could not be detected, 10 were successful TESE (58.2%), and 14 of the 23 cases where AMH was detected were unsuccessful TESE (57.5%), with no difference in SP-AMH levels between successful and unsuccessful TESE NOA men. Similarly, Duvilla et al. [14] found no difference in SP-AMH levels between successful and unsuccessful TESE NOA men. Afterward, a prospective study [17] with 139 NOA men compared SP-AMH concentrations between positive and negative TESE outcomes and concluded that SP-AMH concentrations did not differ significantly between successful and unsuccessful TESE patients. Furthermore, their logistic regression failed to demonstrate the predictive value of SP-AMH for SSR. In summary, SP-AMH cannot currently be used as an effective biomarker for positive sperm extraction.

### Inhibin B

Inhibin B (INHB), a glycoprotein primarily produced by Sertoli cells, has a negative correlation with gonadotropin and FSH in feedback regulation and is closely related to the degree of spermatogenesis in testicular histology [54]. However, the value of serum INHB in predicting focal spermatogenesis remains debatable. Because the majority of INHB secreted by Sertoli cells enters the lumen space, the level of INHB in SP may more accurately reflect the functional state of seminiferous tubules. Nevertheless, there have been few studies on the use of SP-INHB to predict SSR.

Nagata et al. [12] compared SP-INHB concentrations in 17 NOA patients who had successful TESE to 45 NOA patients who had failed TESE and found that the SP-INHB concentrations in the successful TESE group were significantly higher. Further analysis revealed that the best discriminating level was 27.0 pg/mL (sensitivity 88.2%, specificity 93.3%), and multivariate logistic regression analysis revealed that SP-INHB was a significant predictor of TESE outcome. Similarly, Soudabeh et al. [16] showed that the SP-INHB concentrations in the successful TESE NOA men were significantly higher. On the other hand, a preliminary study [14] found no difference in SP-INHB between successful and unsuccessful TESE NOA men (*n* = 26). Another study [17] with a larger sample size compared the concentrations of SP-INHB between 60 NOA patients who had successful TESE and 79 NOA patients who had failed TESE and found that SP-INHB did not differ significantly between the two groups. The area under the receiver operating characteristic curve for SP-INHB was 0.502, and logistic regression did not show SP-INHB predictive value for SSR.

Overall, given the above contradictory results, the predictive value of SP-INHB for SSR in patients with NOA remains debatable. Randomized clinical trials with a large sample size and a multicenter design may help to clarify whether seminal plasma inhibin B contributes to SSR prediction in patients with NOA.

### Other biomarkers

Survivin, which can regulate apoptosis, is found in rodent testes germ cells, particularly mature spermatocytes [55], and a decrease in survivin expression may result in spermatogenesis disorder [56]. Moreover, seminal survivin was detectable in 24 NOA men who had successful TESE but not in 13 NOA men who had unsuccessful TESE [15], implying that seminal survivin was associated with SSR in NOA men. Sertoli cells produce clusterin, which is secreted into the liquid of spermatogenic epithelium and deposited on the membranes of elongated sperm cells and mature sperm [57, 58]. Fukuda et al. [22] found no statistically significant differences of SP-clusterin levels between nine NOA patients who had successful micro-TESE and 19 patients who had failed micro-TESE. However, it is worth noting that their univariate analysis suggested SP-clusterin as a predictor of SSR. Leptin has been linked to spermatogenesis [59]. Ma et al. [18] found a significant difference in SP-leptin levels between positive and negative SSR groups, with a cut-off value of 2.9 ng/mL (sensitivity of 43.1% and specificity of 75.0%) to predict SSR at TESE with a limited value, but using artificial neural networks (ANNs) to combine leptin and other markers can significantly improve the prediction accuracy of sperm retrieval in NOA patients. However, in this study, there were no differences in neutral alpha glucosidase (NAG) and fructose levels between positive and negative TESE NOA patients. Freour et al. [20] used an isotope-coded protein label (ICPL)-based proteomic strategy coupled with a conventional protein assay to identify biomarkers of residual spermatogenesis in SP of NOA men. The results showed that the average concentrations of lectin galactoside binding and soluble 3 binding protein (LGALS3BP) in SP of patients with positive TESE were higher than those in patients with negative TESE, and SP-LGALS3BP concentrations less than 153 ng/mL were correlated with negative TESE results (sensitivity of 100% and specificity of 45%), indicating that SP-LGALS3BP may be an effective predictor of SSR. Furthermore, a number of germ cell-specific moleculars have been discovered to be useful in predicting SSR. By comparing histopathological results and germ cell-specific molecules, Aslani et al. [19] found that the expression of DAZ and PRM2 was related to the results of histopathological examination. There is also evidence that other germ cell-specific moleculars (such as TEX101 [23], ESX1 [21], TNP1, and PRM1 [27]) may help predict SSR, though the evidence is weak.

### Conclusions

Although the predictive value of SP-AMH and SP-INHB for SSR was expected, the available evidence suggests that neither SP-AMH nor SP-INHB is widely accepted as predicting SSR in men with NOA. It is worth noting that RNAs, metabolites, and other biomarkers in SP have demonstrated a high potential in predicting SSR. If the specific role of these RNAs and metabolites in spermatogenesis can be confirmed in future research and further prospective, large sample size, and multicenter trials, it will provide more powerful support for SSR prediction.

## Data Availability

All data generated or analysed during this study are included in this published article and its supplementary information files.

## Abbreviations

AMH: Anti Mullerian hormone
ANNs: Artificial neural networks
ART: Assisted reproductive technology
circRNAs: Circular RNAs
INHB: Inhibin B
LGALS3BP: lectin galactoside binding and soluble 3 binding protein
lncRNAs: Long non-coding RNAs
micro-TESE: Microdissection testicular sperm extraction
miRNAs: MicroRNAs
NAG: Neutral alpha glucosidase
NOA: Non-obstructive azoospermia
NOS: Newcastle-Ottawa scale
OA: Obstructive azoospermia
SP: Seminal plasma
SSR: Successful sperm retrieval
TESE: Conventional testicular sperm extraction
tsRNAs: tRNA derived small RNAs
